# A cross-national comparison of obesity using body mass index-for-age percentiles: results from the Canadian Longitudinal Study on Aging and the United States Health and Retirement Study

**DOI:** 10.1093/aje/kwaf181

**Published:** 2025-08-21

**Authors:** Claire E Cook, Chris Kim, Hailey R Banack

**Affiliations:** Epidemiology Division, Dalla Lana School of Public Health, University of Toronto, Toronto, ON M5T 3M7, Canada; Epidemiology Division, Dalla Lana School of Public Health, University of Toronto, Toronto, ON M5T 3M7, Canada; Epidemiology Division, Dalla Lana School of Public Health, University of Toronto, Toronto, ON M5T 3M7, Canada

**Keywords:** CLSA, obesity, BMI, aging, USA, Canada

## Abstract

Obesity prevalence is increasing in both the United States and Canada concurrently with demographic shifts, resulting in increasingly older populations with a high prevalence of obesity. Older adults have unique risk factors and outcomes related to obesity, such as age-related physiologic changes over time, that need to be considered when assessing obesity. In a comparison of data from the Canadian Longitudinal Study on Aging and the US Health and Retirement Study, we use BMI-for-age percentile curves to examine obesity in the United States and Canada in individuals 50+ years. Overall, BMI values were higher among individuals in the United States and declined with chronological age in both countries. BMI values were higher among women than men in both countries. BMI-for-age percentiles reached a peak at a younger age among women in Canada compared to individuals in the United States. Using a novel measurement of obesity, the present work describes differences in obesity in older adults in Canada and the United States and highlights the need for future work in obesity research in age- and sex-disaggregated contexts.

**This article is part of a Special Collection on Cross-National Gerontology**.

## Introduction

Increasing prevalence of obesity in older adults is a significant clinical and public health concern in both Canada and the United States.^1^ Across North America, population rates of obesity have been increasing since 1980, with prevalence rising more rapidly in recent years.^2^^-^^4^ Among older adults, the prevalence of obesity in the United States from 2017 to 2020 was 41.5% for adults aged 60 and over, using measured body mass index (BMI) from the National Health and Nutrition survey, and in Canada from 2014 to 2015, the prevalence of obesity was 32.8% among adults aged 50-64 and 32.5% among adults aged 65-79, using measured BMI from the Canadian Health Measures Survey.^2^^-^^5^ Obesity prevalence has been increasing over time; in the United States, 23 states had obesity rates of at least 35%, compared to zero states with obesity rates greater than 35% in 2013.^6^^,^^7^ Similarly, in Canada, prevalence of obesity increased from 21% in 2004 to 30% in 2022.^8^ The prevalence of obesity is of particular concern given the presently high burden of obesity among individuals in both the United States and Canada and given the prevalence of obesity has been increasing rapidly over time.

Obesity is associated with an increased burden of chronic disease, a particularly important concern among older adults who are already at a higher risk of morbidity and mortality than the general population.^9^ Both Canada and the United States are experiencing demographic shifts toward aging populations.^10^^,^^11^ For example, 18.5% of the Canadian population is over the age of 65, and 13% are aged 85 or older, compared to 6.7% over the age of 65 in 1966.^10^ Obesity specifically among older adults is a critically understudied area.^12^ Older adults with obesity may have an increased risk for geriatric syndromes, including frailty, sarcopenia, and multimorbidity.^13^^-^^16^ Older adults undergo many physical changes throughout the aging process, which may alter the health effects of obesity.^15^ Further, there are established sex differences in obesity and body composition as individuals age.^17^ It is important to examine obesity within the context of aging and older populations to respond to shifting demographic trends. Descriptive epidemiology is a critical step toward identifying groups in need of clinical or public health intervention.^18^

Limited prior research has directly compared the prevalence of obesity in the United States and Canada. Comparisons between the United States and Canada are of interest given that they are similarly high-income countries that share some social and cultural features. However, the United States and Canada differ on important structural components and social determinants of health (SDOH) that are known to impact health outcomes, including obesity. The United States and Canada have different education and health care systems, access to health care, and public health policies.^17^^-^^21^ The prevalence of obesity in Canada and the United States is driven by a complex interplay of individual level characteristics, as well as social and structural determinants of health. Moreover, the United States is reported to have greater socioeconomic and health disparities, which may contribute to a higher prevalence of obesity.^19^^,^^22^^-^^24^ A cross-national analysis is an opportunity to describe the differences in obesity prevalence as a step toward understanding the different mechanisms and factors that may contribute to obesity among older adults in each country.

Body mass index (kg/m^2^) is commonly used in assessments of obesity prevalence.^25^ A common definition of obesity used is a BMI measurement of ≥30 kg/m^2^, as per the standardized obesity categories outlined by the Centers for Disease Control and Prevention (CDC).^25^ There are known limitations of defining obesity using a BMI cut-point of 30 kg/m^2^, particularly in older adults. The use of a binary obesity cut point may lead to misclassification and incorrect estimates.^26^^,^^27^ For older adults, defining obesity using a binary cut point also may not be sensitive enough to measure important age-related change in BMI. For example, older adults experience many age-related changes, such as loss of lean muscle mass, leading to sarcopenia or frailty. Loss of muscle mass could lead to a lower BMI, or loss of height may lead to a higher BMI; it is therefore possible that among older adults, a higher BMI may be representative of a healthier population and a lower BMI may indicate a less healthy population.^28^ Misclassification of obesity status is therefore a common pitfall among studies of obesity among older adults and may lead to biased estimates.^26^^,^^28^^,^^29^ Recently, Banack and colleagues developed a measurement approach using BMI-for-age percentiles to examine obesity in older adults and account for age-related changes in BMI. This measurement approach is intended to prevent misclassification of obesity status in older adults and promote improved understanding of obesity among an aging population. Moreover, BMI-for-age percentile curves have potentially greater clinical and public health utility, similar to the approach widely used in pediatric populations.^30^^,^^31^ BMI-for-age percentile curves are a novel measurement approach that addresses several methodological challenges in the assessment of obesity.

BMI-for-age percentile curves are a descriptive tool that will facilitate comparisons of obesity among older adults in different populations. The objective of this manuscript is to use BMI-for-age percentile curves to describe and compare obesity in older adults in the United States and Canada. Presenting a cross-national comparison of the descriptive epidemiology of obesity disaggregated by sex may inform future analyses and intervention programs in Canada and the United States.

## Methods

### Study design and population

This is a secondary data analysis of 2 prospective longitudinal cohorts: the Canadian Longitudinal Study on Aging (CLSA) (2011-2018) and the US Health and Retirement Study (HRS) (2010-2018).

The CLSA is a research platform designed to investigate aging trajectories and outcomes in a sample of Canadian adults 45 and older.^32^ At baseline, *n* = 51 338 participants were recruited to participate in the study, divided into a tracking cohort (*n* = 21 241) and comprehensive cohort (*n* = 30 097). Participants in the tracking cohort had data collected by telephone using computer-assisted technology, and participants in the comprehensive cohort had in-home study visits at 11 data collection sites in each of 7 provinces.^32^

The present study will use data from the baseline (2011-2015) and first follow-up visit (2015-2018) of the CLSA comprehensive cohort. The CLSA has a complex sampling design; participants in the comprehensive cohort were recruited from a stratified random sample using provincial health registration databases, random digit dialing of landline telephones, and the Quebec Longitudinal Study on Nutrition and Aging as the sampling frame for the comprehensive cohort.^32^ In the comprehensive cohort, recruits were drawn from individuals living within 25-50 km (depending on the city and accessibility) of 1 of 11 purpose-built data collection centers located in 7 provinces. Use of CLSA data was approved (application ID 2310002).

The HRS is a prospective longitudinal study of older adults (> 50 years) in the United States conducted by the University of Michigan and sponsored by the National Institute on Aging and the Social Security Administration. The HRS began in 1992 and has continued in several waves, utilizing a complex survey design to obtain a nationally representative sample. The HRS involves in-person interviews at baseline with telephone interviews at follow-up every 2 years. The HRS collects comprehensive data on health status, health services use, and economic factors such as income and employment. Physical measures (eg, height, weight, blood pressure) are obtained from a subset of the population at in-person interviews.^33^ The RAND HRS Longitudinal File 2020 (Version 2) public use dataset was used in analyses.^34^ The RAND Longitudinal File is a publicly available dataset that combines 15 HRS waves between 1992 and 2020.^34^ The HRS is sponsored by the National Institute on Aging (grant number NIA U01AG009740) and is conducted by the University of Michigan.

To facilitate comparisons between populations, the CLSA and HRS study populations were restricted to adults aged 50 to 90 years (50 to 89 among females in the CLSA). Given known demographic shifts and population aging, the HRS dataset was limited to Waves 10 to 14 (years 2010 to 2018), so the data are concordant with the same time frame as the CLSA. Individuals with missing data on BMI were removed from analyses.

### Variables

Height and weight are measured at the baseline study visit in both the CLSA and HRS by trained study staff.^32^^,^^33^ Body mass index (kg/m^2^) was calculated from measured height and weight and used in a continuous format. In both the CLSA and HRS, sex was self-reported by participants at the baseline study visit in a binary format (male/female). Race/ethnicity was self-reported by HRS and CLSA participants; we recognize race/ethnicity as a social construct, and the use of race/ethnicity in the context of this work is a proxy for the effects of structural, historic, and systemic racism.^35^^,^^36^

### Statistical analysis

Descriptive statistics are reported for both the CLSA and HRS as mean and standard deviation, median and interquartile range, and frequency and percentage, as appropriate. Body mass index is presented descriptively in the HRS and CLSA as mean, standard deviation, and according to the CDC categorical definitions of obesity.^25^

BMI-for-age percentile curves are used to measure obesity among an older population given the curves incorporate information about BMI, age, and sex together. Percentile estimates were calculated for males and females in 1-year intervals according to age and BMI. Weighted percentile estimates for the 1st, 3rd, 5th, 10th, 25th, 50th, 75th, 85th, 90th, 95th, 97th, and 99th percentiles were calculated for each age group (in years). Canadian Longitudinal Study on Aging and HRS respondent level sample weights were used in all analyses. The reference population used in the CLSA BMI-for-age percentiles is the CLSA baseline (2011-2015) and follow-up visit 1 (2015-2018). The reference population used in the HRS BMI-for-age percentiles is all adults aged 50-90 who participated in Waves 10-14 (2010-2018). The reference population is used for comparison of BMI values to a standard population. Percentile curves for the percentile estimates were generated using locally weighted regression and were fitted using the locally estimated scatterplot smoothing procedure.^37^^,^^38^ Lambda-Mu-Sigma (LMS) method and quantile regression with Box-Cox regression transformation to normality was used to create the final curves.^39^ The Box-Cox power transformation was used to transform raw BMI scores to a normal distribution. The following LMS transformation equations were solved to estimate corresponding *z* scores that are also derived by age in years.^30^^,^^39^


$$ X=M\left(1+ LSZ\right)\hat{\mkern6mu} \left(1/L\right),L\ne 0. $$

$$ X=M\ \exp (SZ),L=0. $$

LMS provides a summary of the changing distribution of the measurement of interest by the 3 curves: lambda for the skewness (*L*), mu for the median (*M*), and sigma for the coefficient of variation (*S*). The 1st, 3rd, 5th, 10th, 25th, 50th, 75th, 85th, 90th, 95th, 97th, and 99th percentiles were plotted with age and BMI according to sex, and BMI values corresponding to *z* scores ±3.0 SD, −2.5 SD, 2.0 SD, 1.5 SD, 1.0 SD, and 0.5 SD were calculated. Consistent with CDC guidelines, individuals with BMI greater than the 85th percentile value are considered overweight, and 95th percentile is considered obese.^25^ In the BMI-for-age plots, these percentiles are referenced via a dashed line. This approach allows for the definition of obesity (BMI > 95th percentile) to change with age. All analyses were conducted using RStudio version July 1, 2022.

## Results

### HRS and CLSA population characteristics

To create the CLSA BMI-for-age percentiles, 56 705 observations (*n* = 29 961 at baseline and *n* = 26 744 at follow-up 1) were used. For the HRS BMI-for-age percentiles, *n* = 35 409 observations were used from study Waves 10-14 (2010-2018). The mean age of the CLSA was 64.1 (±10.2) years, and the mean age of the HRS sample was 66.7 (±10.1) years (Table 1). Both populations consisted primarily of individuals reporting their race as White/Caucasian (CLSA, 71.4%; HRS, 70.5%).

**Table 1:** Demographic characteristics of the CLSA (*n* = 56 705) and HRS cohorts (*n* = 35 409).

**HRS**						
	**Overall (Waves 10-14)**	**Wave 10 (2010)**	**Wave 11 (2012)**	**Wave 12 (2014)**	**Wave 13 (2016)**	**Wave 14 (2018)**
*n*	35 409	7779	7299	6850	7204	6277
Age (mean, SD)	66.7 (10.1)	65.8 (10.4)	66.8 (10.0)	68.2 (9.5)	65.8 (10.4)	67.0 (9.8)
Gender (*n*, %)						
Male	15 100 (42.6)	3327 (42.8)	3152 (43.2)	2916 (42.6)	3059 (42.5)	2646 (42.2)
Female	20 309 (57.4)	4452 (57.2)	4147 (56.8)	3934 (57.4)	4145 (57.5)	3631 (57.8)
Race (*n*, %)						
White/Caucasian	24 971 (70.5)	5661 (72.8)	5283 (72.4)	4969 (72.5)	4848 (67.3)	4210 (67.1)
Black/African American	7038 (19.9)	1485 (19.1)	1405 (19.2)	1298 (18.9)	1520 (21.1)	1330 (21.2)
Other	3300 (9.3)	621 (8.0)	584 (8.0)	572 (8.4)	806 (11.2)	717 (11.4)
Missing	100 (0.3)	12 (0.2)	27 (0.4)	11 (0.2)	30 (0.4)	20 (0.3)
Hispanic (*n*, %)						
Non-Hispanic	30 406 (85.9)	6831 (87.8)	6291 (86.2)	5957 (87.0)	6035 (83.8)	5292 (84.3)
Hispanic	4956 (14.0)	941 (12.1)	995 (13.6)	887 (12.9)	1158 (16.1)	975 (15.5)
Missing	47 (0.1)	7 (0.1)	13 (0.2)	6 (0.1)	11 (0.2)	10 (0.2)
Height (mean, SD, cm)	165.4 (10.2)	165.5 (10.2)	165.5 (10.4)	165.2 (10.0)	165.5 (10.1)	165.3 (10.2)
Weight (mean, SD, kg)	82.3 (18.2)	82.1 (18.0)	81.8 (18.1)	81.9 (18.1)	83.1 (18.4)	82.7 (18.3)
BMI (mean, SD, kg/m^2^)	30.1 (6.1)	29.9 (6.1)	29.8 (6.1)	30.0 (6.1)	30.3 (6.3)	30.3 (6.1)
BMI category (*n*, %)						
Underweight (<18.5 kg/m^2^)	102 (0.3)	15 (0.2)	27 (0.4)	28 (0.4)	17 (0.2)	15 (0.2)
Normal range (18.5-24.9 kg/m^2^)	6947 (19.6)	1546 (19.9)	1540 (21.1)	1355 (19.8)	1348 (18.7)	1158 (18.4)
Overweight (25.0-29.9 kg/m^2^)	12 334 (34.8)	2760 (35.5)	2530 (34.7)	2392 (34.9)	2471 (34.3)	2181 (34.7)
Obese (≥30 kg/m^2^)	16 026 (45.3)	3458 (44.5)	3202 (43.9)	3075 (44.9)	3368 (46.8)	2923 (46.6)
**CLSA**						
	**Overall (2011-2018)**		**Baseline (2011-2015)**		**Follow-up 1 (2015-2018)**	
*n*	56 705		29 961		26 744	
Age (mean, SD)	64.1 (10.2)		63.0 (10.2)		65.4 (10.0)	
Gender (*n*, %)						
Male	27 899 (49.2)		14 711 (49.1)		13 188 (49.3)	
Female	28 806 (50.8)		15 250 (50.9)		13 556 (50.7)	
Race/ethnicity (*n*, %)						
Caucasian	40 493 (71.4)		21 287 (71.0)		19 206 (71.8)	
Asian	654 (1.2)		304 (1.0)		350 (1.3)	
African	261 (0.5)		181 (0.6)		80 (0.3)	
Hispanic	134 (0.2)		81 (0.3)		53 (0.2)	
Other ethnicity	141 (0.2)		52 (0.2)		89 (0.3)	
Missing/skip/not administered	15 022 (26.4)		8056 (26.9)		6966 (26.0)	
Height (cm)	168.3 (9.7)		168.3 (9.7)		168.3 (9.7)	
Weight (kg)	79.8 (17.7)		79.7 (17.6)		79.8 (17.9)	
BMI (kg/m^2^)	28.1 (5.5)		28.1 (5.4)		28.1 (5.5)	
BMI category (*n*, %)						
Underweight (<18.5 kg/m^2^)	395 (0.7)		202 (0.7)		193 (0.7)	
Normal range (18.5-24.9 kg/m^2^)	16 337 (28.8)		8610 (28.7)		7727 (28.9)	
Overweight (25.0-29.9 kg/m^2^)	23 320 (41.1)		12 356 (41.2)		10 964 (41.0)	
Obese (≥30 kg/m^2^)	16 653 (29.4)		8793 (29.3)		7860 (29.4)	

Abbreviations: BMI, body mass index; CLSA, Canadian Longitudinal Study on Aging; HRS, Health and Retirement Study.

### Comparison of obesity between HRS and CLSA participants using standard BMI categories

Among CLSA participants, the overall mean BMI was 28.1 (±5.5) kg/m^2^ (Table 1). Using CDC-defined categories of obesity,^25^ the CLSA population was primarily overweight (25.0-29.9 kg/m^2^, 41.1%) or obese (≥30 kg/m^2^, 29.4%) (Table 1). Among the HRS participants, the overall mean BMI was 30.1 (±5.5) kg/m^2^ and varied from 29.8 (±6.1) to 30.3 (±6.3) between Waves 10 and 14. Most HRS participants overall were overweight (25.0-29.9 kg/m^2^, 34.8%) or obese (≥30 kg/m^2^, 45.3%) (Table 1). HRS participants compared to CLSA participants had a higher mean BMI and a higher proportion of participants in the obese category.

### Comparison of obesity between HRS and CLSA participants using BMI-for-age percentiles

**Figure 1:**
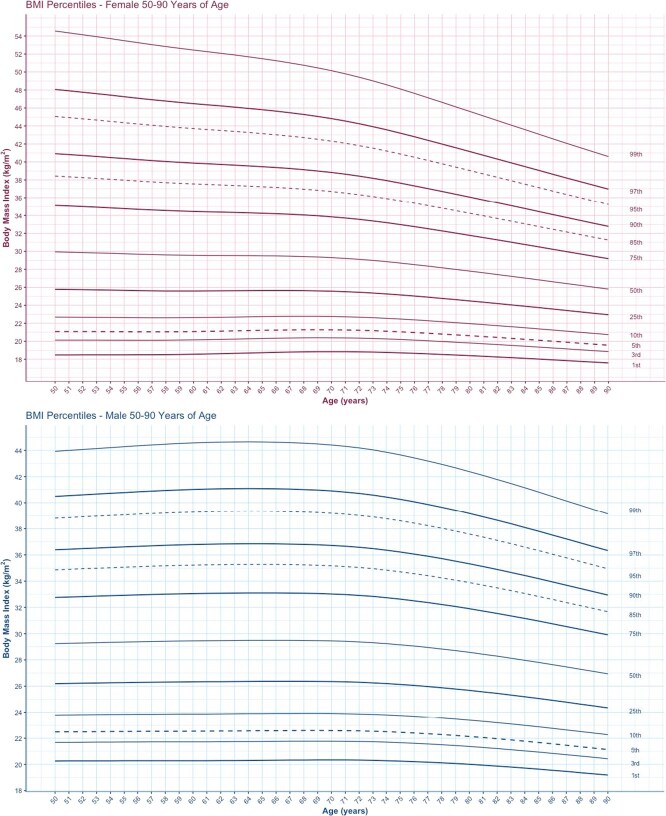
BMI-for-age percentile curves for participants aged 50 to 90 years in the HRS. (A) Females. (B) Males.

**Figure 2:**
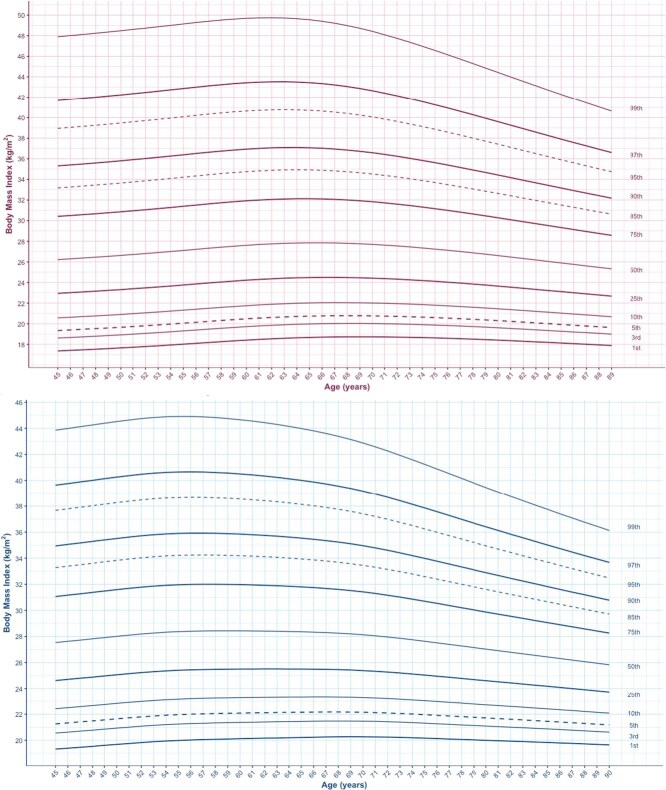
BMI-for-age percentile curves for participants aged 45 to 90 years in the CLSA. (A) Females. (B) Males.

Smoothed BMI-for-age percentile curves are presented for men and women aged 50 to 90 years in the HRS and CLSA (Figures 1 and 2) for the 1st, 3rd, 5th, 10th, 25th, 50th, 75th, 85th, 90th, 95th, 97th, and 99th percentiles. Among females in the HRS, BMI decreased with age, and among males, BMI percentiles increased slightly before decreasing with age. In the HRS, the maximum BMI in the 99th percentiles is 54.6 kg/m^2^ among females at age 50 and 44.6 kg/m^2^ among males at age 61 (Figure 1; Tables S1 and S2). The maximum BMI values for males are lower than females and occur at a later age among males. In the HRS, the BMI value in the 50th percentile is 29.9 among females at age 50 and 29.5 kg/m^2^ among males between ages 65 and 67. There is little variation over time in BMI values among the lowest percentiles (<25th) in both men and women (Figure 1; Tables S1 and S2).

### BMI-for-age percentiles among CLSA participants

Among both males and females in the CLSA, BMI increases slightly before decreasing as chronological age increases. Among CLSA participants, the maximum BMI in the 99th percentiles is nearly 49.7 kg/m^2^ among females at age 62 and 44.9 kg/m^2^ among males at ages 55 and 56 (Figure 2; Tables S3 and S4). Similar to the HRS, the maximum BMI values for males are lower than for females. In contrast to the HRS participants, among CLSA participants, the maximum BMI in the 99th percentile occurs at a later age for females than for males. In the CLSA, the BMI value in the 50th percentile is around 27.9 kg/m^2^ among females between ages 65 and 66 and is 28.4 kg/m^2^ among males between ages 58 and 60 (Figure 2; Tables S3 and S4). In agreement with the HRS population, there is little variation over time in BMI values among the lowest percentiles (<25th) in both men and women (Figure 2).

### Comparison of obesity between HRS and CLSA BMI-for-age percentile *z* scores

BMI *z* scores for males and females change with age in both the HRS and CLSA populations (Tables 2-4). In the HRS, a *z* score of −2.0 corresponds with decreasing BMI values from 21.3 kg/m^2^ at age 50 to 18.7 kg/m^2^ at age 90 among males and with decreasing BMI values from 19.7 kg/m^2^ at age 50 to 18.5 kg/m^2^ at age 90 among females (Tables 2 and 3). In the CLSA, a *z* score of −2.0 corresponds with decreasing BMI values from 20.5 kg/m^2^ at age 50 to 20.4 kg/m^2^ at age 90 among males and with decreasing BMI values from 18.4 kg/m^2^ at age 50 to 18.8 kg/m^2^ at age 89 among females (Table 4).

**Table 2:** Age-specific BMI *z* score values for HRS participants in Waves 10-14 aged 50-90 (*n* = 35 409).

**Age**	**−3.0 SD**	**−2.5 SD**	**−2.0 SD**	**−1.5 SD**	**−1.0 SD**	**−0.5 SD**	**0.5 SD**	**1.0 SD**	**1.5 SD**	**2.0 SD**	**2.5 SD**	**3.0 SD**
Females												
50	16.32	17.89	19.67	21.7	24.05	26.77	33.67	38.08	43.34	49.69	57.42	66.95
51	16.34	17.89	19.67	21.7	24.04	26.75	33.61	37.99	43.22	49.52	57.19	66.63
52	16.35	17.9	19.67	21.7	24.03	26.72	33.55	37.91	43.1	49.35	56.95	66.31
53	16.36	17.91	19.67	21.69	24.01	26.7	33.49	37.82	42.98	49.18	56.72	65.98
54	16.36	17.91	19.67	21.68	23.99	26.66	33.42	37.72	42.85	49	56.47	65.64
55	16.37	17.91	19.66	21.67	23.97	26.63	33.35	37.62	42.71	48.82	56.22	65.3
56	16.38	17.91	19.66	21.66	23.95	26.6	33.28	37.53	42.58	48.63	55.97	64.96
57	16.39	17.92	19.66	21.65	23.94	26.57	33.22	37.44	42.45	48.46	55.73	64.63
58	16.41	17.94	19.67	21.66	23.93	26.55	33.16	37.36	42.33	48.29	55.49	64.3
59	16.43	17.96	19.69	21.66	23.93	26.54	33.12	37.28	42.22	48.13	55.27	63.99
60	16.46	17.98	19.71	21.68	23.94	26.54	33.08	37.22	42.12	47.98	55.05	63.68
61	16.49	18.01	19.74	21.7	23.95	26.54	33.05	37.16	42.03	47.84	54.85	63.38
62	16.53	18.05	19.77	21.73	23.97	26.55	33.02	37.1	41.93	47.7	54.64	63.08
63	16.57	18.09	19.8	21.75	23.99	26.56	32.99	37.05	41.84	47.55	54.42	62.77
64	16.61	18.13	19.84	21.78	24.01	26.56	32.96	36.98	41.74	47.4	54.19	62.44
65	16.66	18.16	19.87	21.81	24.02	26.57	32.92	36.92	41.63	47.23	53.96	62.1
66	16.7	18.2	19.9	21.83	24.03	26.56	32.88	36.84	41.51	47.06	53.7	61.74
67	16.73	18.23	19.92	21.85	24.04	26.56	32.82	36.76	41.38	46.87	53.43	61.36
68	16.76	18.26	19.94	21.86	24.04	26.54	32.76	36.65	41.23	46.66	53.14	60.96
69	16.79	18.28	19.95	21.86	24.03	26.51	32.68	36.54	41.06	46.42	52.82	60.52
70	16.8	18.29	19.96	21.85	24	26.46	32.58	36.4	40.87	46.16	52.46	60.04
71	16.81	18.28	19.94	21.82	23.96	26.41	32.46	36.23	40.66	45.87	52.08	59.53
72	16.81	18.27	19.92	21.79	23.91	26.33	32.32	36.05	40.41	45.55	51.66	58.99
73	16.8	18.25	19.89	21.74	23.84	26.24	32.16	35.84	40.14	45.21	51.21	58.4
74	16.78	18.22	19.84	21.68	23.76	26.13	31.98	35.61	39.85	44.83	50.73	57.79
75	16.74	18.18	19.78	21.6	23.66	26.01	31.78	35.36	39.54	44.43	50.23	57.14
76	16.71	18.13	19.72	21.52	23.56	25.87	31.57	35.1	39.21	44.02	49.7	56.47
77	16.66	18.07	19.65	21.43	23.44	25.73	31.35	34.83	38.86	43.59	49.16	55.79
78	16.62	18.01	19.57	21.33	23.32	25.59	31.13	34.55	38.52	43.16	48.62	55.11
79	16.57	17.95	19.5	21.24	23.2	25.44	30.9	34.26	38.17	42.72	48.07	54.42
80	16.52	17.89	19.42	21.14	23.08	25.28	30.67	33.98	37.81	42.28	47.53	53.73
81	16.47	17.82	19.34	21.04	22.96	25.13	30.44	33.69	37.46	41.84	46.98	53.05
82	16.41	17.75	19.25	20.93	22.83	24.97	30.2	33.4	37.1	41.4	46.43	52.37
83	16.36	17.68	19.16	20.83	22.7	24.81	29.96	33.11	36.74	40.96	45.89	51.69
84	16.3	17.61	19.07	20.72	22.56	24.65	29.72	32.81	36.38	40.51	45.34	51.01
85	16.24	17.53	18.98	20.6	22.43	24.48	29.47	32.51	36.02	40.07	44.79	50.34
86	16.17	17.45	18.89	20.49	22.29	24.32	29.23	32.22	35.65	39.63	44.25	49.67
87	16.11	17.37	18.79	20.37	22.15	24.15	28.98	31.92	35.29	39.19	43.71	49.01
88	16.04	17.29	18.69	20.25	22.01	23.98	28.74	31.62	34.93	38.75	43.18	48.36
89	15.97	17.21	18.59	20.14	21.86	23.81	28.49	31.33	34.58	38.32	42.66	47.72
90	15.9	17.13	18.5	20.02	21.72	23.64	28.25	31.04	34.23	37.9	42.14	47.09
Males												
50	18.34	19.74	21.29	22.99	24.87	26.94	31.8	34.64	37.82	41.38	45.37	49.86
51	18.34	19.75	21.29	23	24.88	26.96	31.83	34.68	37.87	41.44	45.44	49.95
52	18.34	19.75	21.3	23.01	24.89	26.98	31.86	34.72	37.92	41.5	45.52	50.05
53	18.34	19.75	21.3	23.01	24.9	26.99	31.89	34.76	37.97	41.56	45.6	50.14
54	18.34	19.75	21.31	23.02	24.92	27.01	31.92	34.8	38.02	41.62	45.67	50.23
55	18.34	19.75	21.31	23.03	24.93	27.03	31.95	34.84	38.07	41.68	45.75	50.33
56	18.33	19.75	21.32	23.04	24.94	27.05	31.98	34.88	38.11	41.74	45.82	50.42
57	18.33	19.76	21.32	23.04	24.95	27.06	32.01	34.91	38.16	41.8	45.89	50.5
58	18.33	19.75	21.32	23.05	24.96	27.07	32.03	34.94	38.2	41.85	45.95	50.58
59	18.33	19.75	21.32	23.05	24.97	27.08	32.05	34.97	38.23	41.89	46.01	50.65
60	18.33	19.75	21.32	23.06	24.97	27.09	32.07	34.99	38.26	41.93	46.06	50.71
61	18.33	19.75	21.33	23.06	24.98	27.1	32.08	35.01	38.29	41.96	46.1	50.76
62	18.33	19.76	21.33	23.07	24.98	27.11	32.1	35.03	38.31	41.98	46.12	50.79
63	18.33	19.76	21.34	23.07	24.99	27.12	32.11	35.04	38.32	42	46.14	50.81
64	18.34	19.77	21.34	23.08	25	27.13	32.11	35.05	38.33	42	46.14	50.81
65	18.35	19.78	21.35	23.09	25.01	27.13	32.12	35.05	38.32	42	46.13	50.8
66	18.36	19.79	21.36	23.1	25.01	27.14	32.12	35.04	38.31	41.98	46.11	50.77
67	18.37	19.8	21.37	23.1	25.02	27.14	32.11	35.03	38.3	41.96	46.08	50.72
68	18.38	19.81	21.37	23.11	25.02	27.13	32.1	35.01	38.27	41.92	46.03	50.66
69	18.39	19.81	21.38	23.11	25.01	27.13	32.08	34.98	38.23	41.87	45.97	50.58
70	18.39	19.81	21.38	23.1	25	27.11	32.05	34.94	38.18	41.81	45.88	50.48
71	18.39	19.81	21.37	23.09	24.99	27.09	32	34.89	38.11	41.72	45.78	50.34
72	18.39	19.8	21.36	23.07	24.96	27.05	31.95	34.82	38.02	41.61	45.64	50.18
73	18.38	19.79	21.34	23.04	24.92	27	31.87	34.73	37.91	41.48	45.48	49.99
74	18.36	19.77	21.31	23	24.88	26.95	31.79	34.62	37.78	41.32	45.29	49.76
75	18.34	19.74	21.27	22.96	24.82	26.88	31.68	34.49	37.63	41.14	45.08	49.5
76	18.31	19.7	21.23	22.9	24.75	26.79	31.56	34.35	37.46	40.94	44.84	49.22
77	18.28	19.66	21.17	22.84	24.68	26.7	31.44	34.2	37.28	40.73	44.58	48.91
78	18.24	19.61	21.12	22.77	24.6	26.61	31.3	34.04	37.09	40.5	44.31	48.59
79	18.2	19.56	21.06	22.7	24.51	26.5	31.15	33.86	36.88	40.25	44.02	48.25
80	18.15	19.51	20.99	22.62	24.41	26.39	30.99	33.68	36.67	40	43.72	47.9
81	18.1	19.45	20.92	22.53	24.31	26.27	30.83	33.49	36.44	39.73	43.41	47.53
82	18.05	19.38	20.84	22.44	24.2	26.14	30.66	33.28	36.2	39.46	43.09	47.16
83	17.99	19.31	20.76	22.35	24.09	26.01	30.48	33.08	35.96	39.17	42.76	46.77
84	17.93	19.24	20.67	22.25	23.97	25.88	30.29	32.86	35.71	38.88	42.42	46.37
85	17.86	19.16	20.58	22.14	23.85	25.73	30.1	32.64	35.45	38.58	42.07	45.97
86	17.79	19.08	20.49	22.03	23.73	25.59	29.91	32.41	35.19	38.28	41.72	45.56
87	17.72	18.99	20.39	21.92	23.6	25.44	29.71	32.18	34.93	37.97	41.36	45.15
88	17.64	18.91	20.29	21.8	23.46	25.29	29.51	31.95	34.66	37.67	41.01	44.74
89	17.57	18.82	20.19	21.69	23.33	25.14	29.31	31.72	34.39	37.36	40.66	44.33
90	17.49	18.73	20.09	21.57	23.2	24.98	29.1	31.49	34.13	37.05	40.3	43.93

Abbreviations: BMI, body mass index; HRS, Health and Retirement Study.

**Table 3:** Age-specific BMI *z* score values for CLSA participants aged 47-89 (*n* = 56 705).

**Age (years)**	**−3.0 SD**	**−2.5 SD**	**−2.0 SD**	**−1.5 SD**	**−1.0 SD**	**−0.5 SD**	**0.5 SD**	**1.0 SD**	**1.5 SD**	**2.0 SD**	**2.5 SD**	**3.0 SD**
Females												
47	15.7	16.9	18.3	19.8	21.6	23.7	29.2	32.9	37.4	43.2	50.8	61.0
48	15.8	17.0	18.3	19.9	21.7	23.8	29.3	33.0	37.5	43.3	50.9	61.1
49	15.8	17.0	18.4	19.9	21.8	23.9	29.4	33.1	37.6	43.4	51.0	61.3
50	15.9	17.1	18.4	20.0	21.8	23.9	29.5	33.2	37.7	43.5	51.1	61.4
51	15.9	17.1	18.5	20.1	21.9	24.0	29.6	33.3	37.8	43.7	51.2	61.5
52	16.0	17.2	18.6	20.1	22.0	24.1	29.7	33.4	37.9	43.8	51.4	61.6
53	16.1	17.3	18.6	20.2	22.0	24.2	29.8	33.5	38.1	43.9	51.5	61.8
54	16.1	17.3	18.7	20.3	22.1	24.3	29.9	33.6	38.2	44.0	51.6	61.9
55	16.2	17.4	18.8	20.4	22.2	24.4	30.0	33.7	38.3	44.1	51.8	62.0
56	16.3	17.5	18.9	20.5	22.3	24.5	30.1	33.8	38.4	44.3	51.9	62.2
57	16.3	17.6	19.0	20.5	22.4	24.6	30.2	33.9	38.5	44.4	52.0	62.3
58	16.4	17.6	19.0	20.6	22.5	24.7	30.3	34.0	38.7	44.5	52.2	62.5
59	16.5	17.7	19.1	20.7	22.6	24.8	30.4	34.2	38.8	44.7	52.3	62.6
60	16.6	17.8	19.2	20.8	22.7	24.9	30.5	34.3	38.9	44.8	52.4	62.7
61	16.6	17.9	19.3	20.9	22.8	24.9	30.6	34.4	39.0	44.9	52.5	62.7
62	16.7	17.9	19.4	21.0	22.8	25.0	30.7	34.5	39.1	45.0	52.6	62.8
63	16.8	18.0	19.4	21.0	22.9	25.1	30.8	34.5	39.2	45.0	52.6	62.8
64	16.8	18.1	19.5	21.1	23.0	25.2	30.8	34.6	39.2	45.1	52.6	62.7
65	16.9	18.1	19.5	21.2	23.0	25.2	30.9	34.6	39.2	45.1	52.6	62.6
66	16.9	18.2	19.6	21.2	23.1	25.3	30.9	34.6	39.2	45.0	52.5	62.4
67	17.0	18.2	19.6	21.2	23.1	25.3	30.9	34.6	39.2	44.9	52.4	62.2
68	17.0	18.2	19.6	21.3	23.1	25.3	30.9	34.6	39.1	44.8	52.2	61.9
69	17.0	18.3	19.7	21.3	23.1	25.3	30.9	34.5	39.0	44.7	52.0	61.6
70	17.0	18.3	19.7	21.3	23.1	25.3	30.8	34.5	38.9	44.5	51.7	61.2
71	17.0	18.3	19.7	21.3	23.1	25.2	30.8	34.4	38.8	44.3	51.4	60.7
72	17.0	18.3	19.7	21.3	23.1	25.2	30.7	34.2	38.6	44.1	51.1	60.2
73	17.0	18.3	19.6	21.2	23.0	25.2	30.6	34.1	38.4	43.8	50.7	59.7
74	17.0	18.3	19.6	21.2	23.0	25.1	30.5	34.0	38.2	43.5	50.3	59.1
75	17.0	18.2	19.6	21.2	23.0	25.0	30.3	33.8	38.0	43.2	49.9	58.5
76	17.0	18.2	19.6	21.1	22.9	25.0	30.2	33.6	37.8	42.9	49.4	57.9
77	17.0	18.2	19.5	21.1	22.8	24.9	30.1	33.4	37.5	42.6	49.0	57.3
78	17.0	18.1	19.5	21.0	22.8	24.8	29.9	33.2	37.3	42.2	48.5	56.6
79	16.9	18.1	19.4	21.0	22.7	24.7	29.8	33.0	37.0	41.9	48.0	56.0
80	16.9	18.1	19.4	20.9	22.6	24.6	29.6	32.8	36.7	41.5	47.6	55.3
81	16.9	18.0	19.3	20.8	22.5	24.5	29.4	32.6	36.5	41.2	47.1	54.7
82	16.8	18.0	19.3	20.8	22.4	24.4	29.3	32.4	36.2	40.8	46.6	54.0
83	16.8	17.9	19.2	20.7	22.3	24.3	29.1	32.2	35.9	40.4	46.1	53.3
84	16.7	17.9	19.2	20.6	22.2	24.1	28.9	32.0	35.6	40.1	45.6	52.7
85	16.7	17.8	19.1	20.5	22.2	24.0	28.7	31.7	35.3	39.7	45.2	52.1
86	16.7	17.8	19.0	20.4	22.1	23.9	28.6	31.5	35.1	39.4	44.7	51.4
87	16.6	17.7	19.0	20.4	22.0	23.8	28.4	31.3	34.8	39.0	44.2	50.8
88	16.6	17.7	18.9	20.3	21.9	23.7	28.2	31.1	34.5	38.7	43.8	50.2
89	16.5	17.6	18.8	20.2	21.8	23.6	28.0	30.9	34.2	38.3	43.3	49.6
Males												
46	17.7	18.9	20.2	21.7	23.4	25.3	30.1	33.1	36.5	40.7	45.7	51.9
47	17.8	18.9	20.3	21.8	23.5	25.4	30.2	33.2	36.7	40.8	45.9	52.0
48	17.8	19.0	20.4	21.9	23.6	25.5	30.3	33.3	36.8	41.0	46.0	52.2
49	17.9	19.1	20.4	21.9	23.7	25.6	30.4	33.4	36.9	41.1	46.1	52.3
50	18.0	19.2	20.5	22.0	23.7	25.7	30.5	33.5	37.0	41.2	46.3	52.5
51	18.0	19.2	20.6	22.1	23.8	25.8	30.6	33.6	37.1	41.3	46.4	52.6
52	18.1	19.3	20.7	22.2	23.9	25.9	30.7	33.7	37.2	41.5	46.5	52.7
53	18.2	19.4	20.7	22.3	24.0	25.9	30.8	33.8	37.3	41.6	46.6	52.8
54	18.2	19.4	20.8	22.3	24.1	26.0	30.9	33.9	37.4	41.6	46.7	52.9
55	18.3	19.5	20.9	22.4	24.1	26.1	30.9	33.9	37.5	41.7	46.8	52.9
56	18.3	19.5	20.9	22.4	24.2	26.1	31.0	34.0	37.5	41.7	46.8	53.0
57	18.3	19.6	20.9	22.5	24.2	26.2	31.0	34.0	37.5	41.7	46.8	52.9
58	18.4	19.6	21.0	22.5	24.2	26.2	31.0	34.0	37.5	41.7	46.7	52.9
59	18.4	19.6	21.0	22.5	24.2	26.2	31.0	34.0	37.5	41.7	46.7	52.8
60	18.4	19.6	21.0	22.5	24.3	26.2	31.0	34.0	37.5	41.6	46.6	52.6
61	18.4	19.7	21.0	22.5	24.3	26.2	31.0	34.0	37.4	41.6	46.5	52.5
62	18.5	19.7	21.0	22.6	24.3	26.2	31.0	33.9	37.4	41.5	46.4	52.3
63	18.5	19.7	21.1	22.6	24.3	26.2	31.0	33.9	37.3	41.4	46.2	52.1
64	18.5	19.7	21.1	22.6	24.3	26.2	30.9	33.8	37.2	41.3	46.1	51.9

**Table 3:** Continued

**Age (years)**	**−3.0 SD**	**−2.5 SD**	**−2.0 SD**	**−1.5 SD**	**−1.0 SD**	**−0.5 SD**	**0.5 SD**	**1.0 SD**	**1.5 SD**	**2.0 SD**	**2.5 SD**	**3.0 SD**
65	18.5	19.7	21.1	22.6	24.3	26.2	30.9	33.8	37.2	41.2	45.9	51.7
66	18.6	19.8	21.1	22.6	24.3	26.2	30.8	33.7	37.1	41.0	45.7	51.4
67	18.6	19.8	21.1	22.6	24.3	26.2	30.8	33.7	37.0	40.9	45.5	51.1
68	18.6	19.8	21.1	22.6	24.3	26.2	30.7	33.6	36.9	40.7	45.3	50.8
69	18.6	19.8	21.1	22.6	24.3	26.1	30.7	33.5	36.7	40.5	45.1	50.5
70	18.6	19.8	21.1	22.6	24.3	26.1	30.6	33.4	36.6	40.3	44.8	50.1
71	18.7	19.8	21.1	22.6	24.2	26.1	30.5	33.2	36.4	40.1	44.5	49.7
72	18.7	19.8	21.1	22.6	24.2	26.0	30.4	33.1	36.2	39.9	44.2	49.3
73	18.7	19.8	21.1	22.5	24.1	25.9	30.3	32.9	36.0	39.6	43.8	48.9
74	18.6	19.8	21.1	22.5	24.1	25.8	30.1	32.8	35.8	39.3	43.5	48.4
75	18.6	19.8	21.0	22.4	24.0	25.8	30.0	32.6	35.6	39.0	43.1	47.9
76	18.6	19.7	21.0	22.4	23.9	25.7	29.9	32.4	35.3	38.7	42.7	47.4
77	18.6	19.7	21.0	22.3	23.9	25.6	29.7	32.2	35.1	38.4	42.3	46.9
78	18.6	19.7	20.9	22.3	23.8	25.5	29.6	32.0	34.8	38.1	41.9	46.4
79	18.6	19.7	20.9	22.2	23.7	25.4	29.4	31.8	34.6	37.8	41.5	45.9
80	18.5	19.6	20.8	22.2	23.6	25.3	29.2	31.6	34.4	37.5	41.1	45.4
81	18.5	19.6	20.8	22.1	23.6	25.2	29.1	31.4	34.1	37.2	40.8	44.9
82	18.5	19.6	20.8	22.1	23.5	25.1	28.9	31.2	33.9	36.9	40.4	44.5
83	18.5	19.5	20.7	22.0	23.4	25.0	28.8	31.1	33.6	36.6	40.0	44.0
84	18.5	19.5	20.7	21.9	23.4	24.9	28.6	30.9	33.4	36.3	39.6	43.5
85	18.4	19.5	20.6	21.9	23.3	24.8	28.5	30.7	33.2	36.0	39.3	43.1
86	18.4	19.5	20.6	21.8	23.2	24.7	28.3	30.5	32.9	35.7	38.9	42.6
87	18.4	19.4	20.5	21.8	23.1	24.6	28.2	30.3	32.7	35.4	38.6	42.2
88	18.4	19.4	20.5	21.7	23.1	24.5	28.0	30.1	32.5	35.1	38.2	41.7
89	18.4	19.4	20.5	21.7	23.0	24.4	27.9	29.9	32.2	34.9	37.9	41.3
90	18.3	19.3	20.4	21.6	22.9	24.3	27.7	29.8	32.0	34.6	37.5	40.9

Abbreviations: BMI, body mass index; CLSA, Canadian Longitudinal Study on Aging.

**Table 4:** Comparison of HRS and CLSA BMI percentiles according to sex.

	**10th**	**25th**	**50th**	**75th**	**99th**	**1 SD**	**2 SD**	**3 SD**
Females								
Age 50								
HRS	22.69	25.78	29.94	35.12	54.56	38.08	49.69	66.95
CLSA	20.90	23.31	26.63	30.86	48.48	33.20	43.50	61.40
Age 75								
HRS	22.47	25.15	28.69	32.97	48.10	35.36	44.43	57.14
CLSA	21.80	24.12	27.24	31.16	46.57	33.80	43.20	58.50
Age 89^a^								
HRS	20.87	23.10	26.0	29.44	41.08	31.33	38.32	47.72
CLSA	20.68	22.68	25.33	28.58	40.65	30.39	38.30	49.60
Males								
Age 50								
HRS	23.79	26.19	29.24	32.76	43.93	34.64	41.38	49.86
CLSA	22.83	26.06	28.01	31.59	44.52	33.50	41.20	52.50
Age 75								
HRS	23.75	26.13	29.15	32.63	43.66	34.49	41.14	49.50
CLSA	23.04	25.04	27.63	30.70	41.24	32.60	39.0	47.90
Age 90								
HRS	22.27	24.34	26.94	29.91	39.14	31.49	37.05	43.93
CLSA	22.08	23.73	25.83	28.26	36.13	29.80	34.60	40.90

Abbreviations: BMI, body mass index; CLSA: Canadian Longitudinal Study; HRS: Health and Retirement Study.

a Age 90 excluded due to small sample size in the CLSA.

## Discussion

In this manuscript, we present a comparison of BMI-for-age percentiles in the United States and Canada using data from the CLSA and HRS. We used a recently developed approach to create BMI-for-age percentiles for older adults. This accounts for age-related changes in BMI. Overall, the BMI value in the 50th percentile is higher among females than males in both the HRS and CLSA, before declining as age increases. In both the HRS and CLSA, maximum BMI values are also higher among females compared to males. However, maximum BMI values in the HRS population occur at a younger age among female HRS participants compared to female CLSA participants. The CLSA BMI-for-age percentile values are lower than the HRS BMI-for-age among both males and females.

Our findings are consistent with prior literature that has found a higher prevalence of obesity among older adults in the United States compared to Canada.^9^^,^^22^^,^^40^ In a comparison of CLSA and HRS data, Hernández et al.^40^ documented higher self-reported BMI among older adults in the United States (33%) compared to Canada. The authors hypothesized that socioeconomic gradients, differences in health systems, and health policies may contribute to the cross-national differences in obesity. Siddiqi et al.^22^ examined obesity between the United States and Canada specifically in the context of socioeconomic inequalities using the 2002/2003 Joint Canada/U.S. Survey of Health and also found higher prevalence of obesity in the United States in all but the college educated groups. This work did not specifically examine older populations, but adults of any age.

In our study, we found BMI-for-age percentiles were higher in Americans than in Canadians. One potential explanation for the observed difference is related to structural factors within each country, including socioeconomic inequalities and health disparities. The United States and Canada have notable differences in access to health care, behavioral health factors, and SDOH.^19^^,^^20^^,^^41^^,^^42^ Zajacova et al.^19^ examined differences in socioeconomic health inequality between the United States and Canada and found greater socioeconomic equality in the United States compared to Canada. Underlying health status of each nation and the discrepancy between the 2 nations in socioeconomic factors that contribute to the development of obesity may contribute to observed differences in obesity. For example, socioeconomic factors that may impact obesity prevalence include one’s access to adequate health care, ability to purchase healthy food, availability of healthy food in a particular geographic area, lack of access to safe areas for physical activity, and prohibitive costs of pharmacologic treatments or surgery based on insurance type or income level.^21^^,^^23^^,^^43^ Health outcomes like obesity, particularly in late life, depend on the social, political, and economic context in which individuals are aging.^44^^-^^46^ There is a paucity of cross-national comparisons that specifically assess social, economic, and behavioral factors related to obesity among aging populations. A lack of age-disaggregated research may miss the unique considerations necessary when examining obesity among older adults. Future cross-national comparisons among older adults in the United States and Canada should consider socioeconomic and behavioral factors that may contribute to the observed differences in BMI.

The finding that females in both countries had higher BMI values highlights the importance of considering sex differences in obesity as individuals age. Sex differences in the prevalence of obesity are well-established, with women being more likely to be obese than men.^17^ These differences become more pronounced within the context of aging, as women undergo many pathophysiologic changes during the menopausal transition that contribute to increased abdominal adipose tissue, changing distribution of visceral and subcutaneous fat, and loss of muscle mass.^47^^-^^49^ Obesity screening, identification, and prevention may therefore be of particular importance for postmenopausal women in both the United States and Canada. Body mass index percentile curves differed slightly between females in the United States and Canada, with individuals in Canada reaching a peak BMI at a later age than individuals in the United States. Further research is needed to investigate the factors that may contribute to this difference and to identify the optimal timing of clinical and public health intervention for postmenopausal women in each country.

There are several strengths and limitations in the present work. First, both the CLSA and the HRS use complex survey designs and provide sample weights for analysis to obtain nationally representative estimates. The use of BMI-for-age percentiles to describe obesity among an older population accounts for age-related change in BMI that may have otherwise led to misclassification of obesity status and permits a more accurate description of weight status in older adults. A limitation of the development of BMI-for-age percentiles using the CLSA and HRS is that the reference populations in these cohorts are predominately White/Caucasian populations. This may have important considerations for the generalizability of these findings. There are known disparities in obesity prevalence among racial and ethnic groups,^45^ and future work should aim to address this gap. Finally, an inherent limitation of research among older adults is the potential for selection bias introduced by selective survival; inclusion in the HRS and CLSA cohorts requires that individuals have survived until the measurement of BMI in late life.

Obesity among older adults is a critically important, yet understudied, area of epidemiologic research. The confluence of the aging population and increasing prevalence of obesity represents an important threat to public health in Canada and the United States. This descriptive work highlights the importance of measuring obesity using an approach that simultaneously considers age-related change and sex differences. There are established differences in social, cultural, and economic factors between the United States and Canada, and future work should examine obesity among older adults in relation to socioeconomic and behavioral factors that may underlie differences in obesity. This use of BMI-for-age percentile curves also highlights the need for further cross-national comparisons to examine the links between obesity, socio-contextual factors, and geriatric health outcomes worldwide.

## Supplementary Material

Web_Material_kwaf181

## Data Availability

Data are available from the CLSA (www.clsa-elcv.ca) for researchers who meet the criteria for access to de-identified CLSA data. Data are available for the HRS public use dataset online: https://www.rand.org/well-being/social-and-behavioral-policy/portfolios/aging-longevity/dataprod/hrs-data.html.
