# Supplementary material for: A cross-national comparison of obesity using body mass index-for-age percentiles: results from the Canadian Longitudinal Study on Aging and the United States Health and Retirement Study
Source: Am J Epidemiol. 2025 Aug 21;194(12):3693–704. doi: 10.1093/aje/kwaf181 (PMC12671972; doi:10.1093/aje/kwaf181)
Supplement: Web_Material_kwaf181 [file web_material_kwaf181.docx]

**Title:** A Spatial Framework for selecting sentinel sites for Drug and Alcohol related Early Warning for the United States.

Authors: : Suparna Das, PhD; DeLayna Goulding, MPH; Kacie Rubalcava, PhD; Kathleen Aarvig, MPH

Supplementary materials included.

Figure S1, Figure S2ab, Table S1

Figure S1. Scree Plot of Eigenvalues from Principal Component Analysis


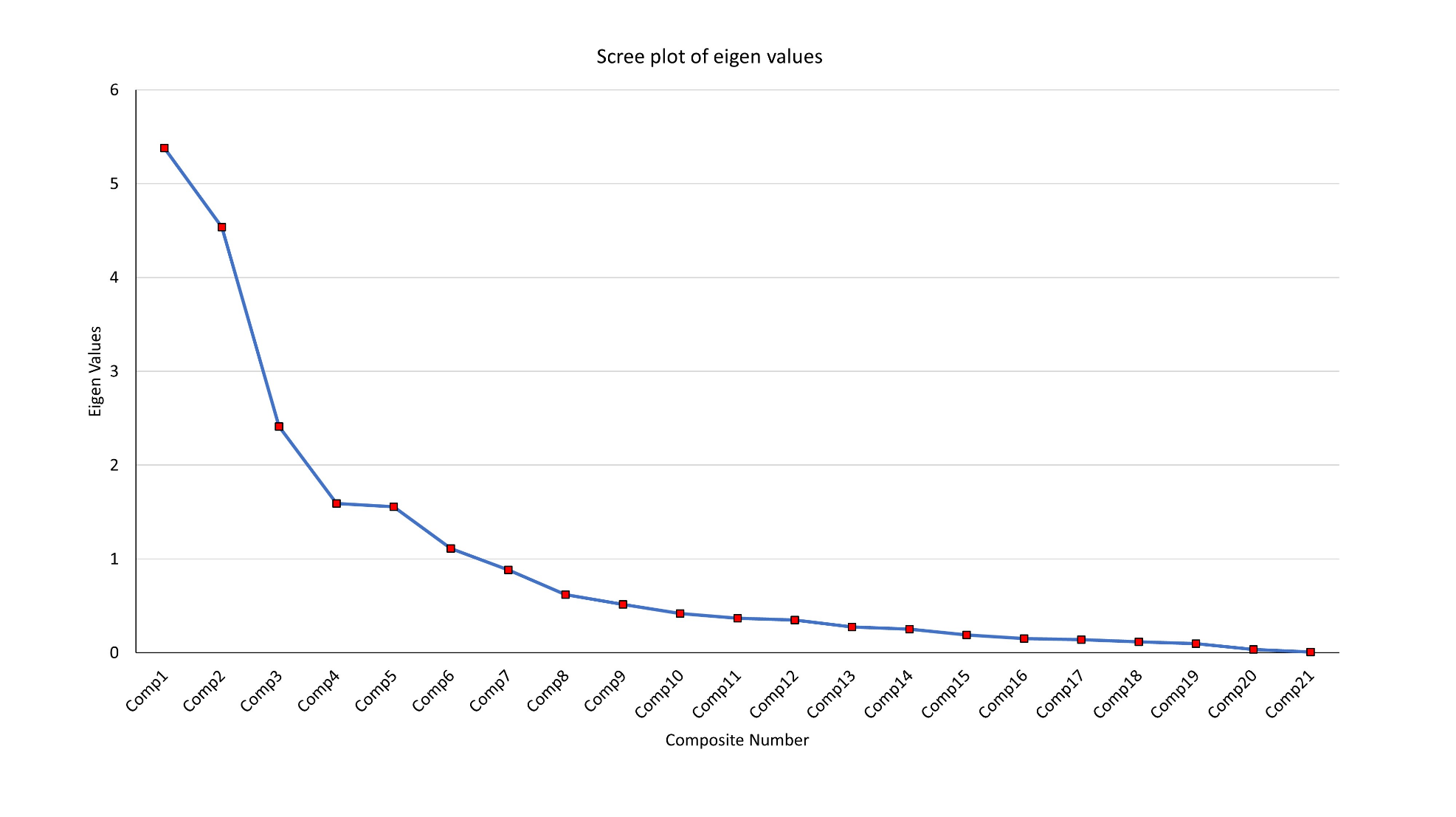


Figure S2. (a) County-level distribution of the total number of DAWN-eligible facilities. Counties shaded dark green do not have any eligible facilities. (b) County-level distribution of emergency department (ED) visits from DAWN-eligible facilities.


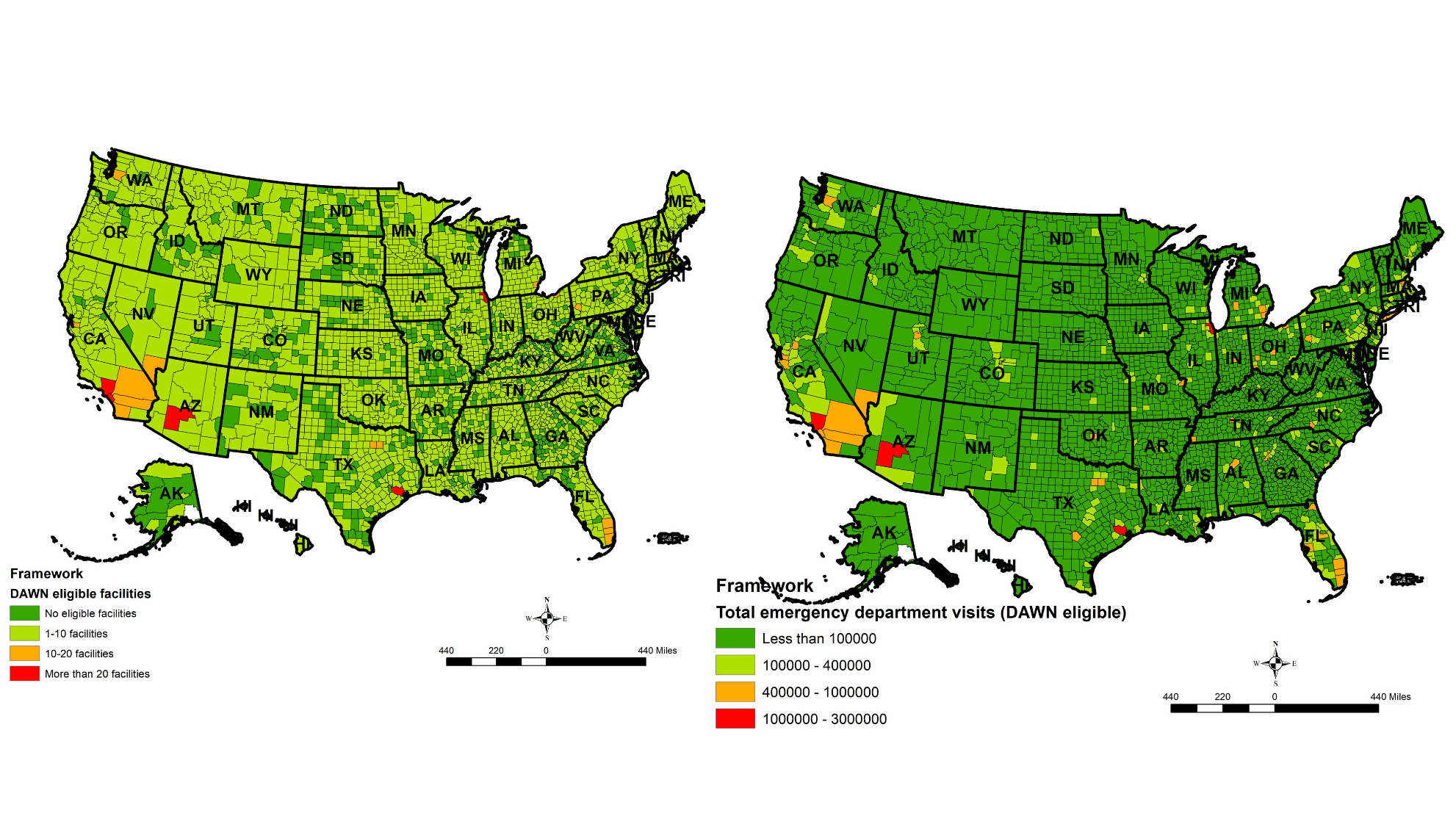


Table S1. Covariance Matrix of standardized variables used in the Principal Component Analysis.
